# A Support System for Adolescent and Young Adult Patients with Cancer at a Comprehensive Cancer Center

**DOI:** 10.31662/jmaj.2021-0106

**Published:** 2021-12-15

**Authors:** Hiroto Ishiki, Takatoshi Hirayama, Saki Horiguchi, Ikumi Iida, Tamae Kurimoto, Mihoko Asanabe, Miho Nakajima, Akiko Sugisawa, Ayako Mori, Yuki Kojima, Ryoko Udagawa, Hayato Tsuchiya, Mami Oki, Mariko Shimizu, Yuko Yanai, Shoko Touma, Keiko Nozawa, Rebekah Kojima, Naoko Inamura, Asami Maehara, Tatsuya Suzuki, Eriko Satomi, On behalf of the AYA Support Team, NCCH

**Affiliations:** 1 Department of Palliative Medicine, National Cancer Center Hospital, Tokyo, Japan; 2 Department of Psycho-Oncology, National Cancer Center Hospital, Tokyo, Japan; 3 Department of Nursing, National Cancer Center Hospital, Tokyo, Japan; 4 Department of Pediatric Oncology, National Cancer Center Hospital, Tokyo, Japan; 5 Department of Medical Oncology, National Cancer Center Hospital, Tokyo, Japan; 6 Department of Pharmacy, National Cancer Center Hospital, Tokyo, Japan; 7 Nutrition Management Office, National Cancer Center Hospital, Tokyo, Japan; 8 Department of Rehabilitation, National Cancer Center Hospital, Tokyo, Japan; 9 Center for Physician Referral and Medical Social Service, National Cancer Center Hospital, Tokyo, Japan; 10 Appearance Support Center, National Cancer Center Hospital, Tokyo, Japan; 11 Department of Hematology, National Cancer Center Hospital, Tokyo, Japan

**Keywords:** Adolescents and young adults, Cancer, Palliative care, Supportive care

## Abstract

Cancer patients in adolescents and young adults (AYA) generation aged 15-39 years have various psychosocial needs during their treatment course such as school enrollment, finding employment, marriage, and fertility. It is difficult for medical professionals to gain experience related to providing medical care and consultation support to these kinds of AYA generation cancer patients. There is a need to provide information and establish both support and medical care systems that are able to meet the diverse needs unique to this generation. This review will explain how to launch an AYA support team (AST).

We have worked and established the AST since 2016, which is medical care teams that provide support according to the life stage of each individual patient and build a multidisciplinary AYA generation patient support system. The team-building process consisted of two main projects: building and enlarging multidisciplinary team and establishing screening process of psychosocial needs of AYA generation patients. Multidisciplinary healthcare professionals got involved in the AST with already-existing patient support functions in our center: the patient support center, which is an outpatient department and the palliative care team, which is an inpatient interdepartmental team. The AST systematically finds patients in need of assistance and offers them support as a multidisciplinary team. The AST also established a procedure that systematically gathers information about the needs of patients by using a screening tool. In addition, the AST provides the following specialized services: reproductive medicine, supporting cancer patients with children, employment support, and peer support. The AST has been established and sophisticatedly worked. It can flexibly provide various psychosocial support services. This review will explain how to launch an AST.

## Introduction

In Japan, about 20,000 people in the adolescents and young adults (AYA) generation (aged 15-39 years old) are diagnosed with cancer annually, and more than 90% of them are 20 years old or older ^(1)^. The types of cancer vary depending on the age group. Between the ages of 15 and 19, there are many rare cancers such as leukemia, lymphoma, sarcoma, and brain tumors. In patients in their 20s, the frequency of these types of cancers decreases, and in patients in their 30s, breast, cervical, and gastrointestinal cancer become more prevalent (Table 1) ^(2), (3)^. The 5-year survival rate for all AYA patients has improved in recent years ^(4)^, and some cancers have 5-year survival rates of greater than 90%, while others have not experienced any change in treatment outcomes ^(5)^.

**Table 1.** Distribution of Cancer Types Among Adolescents and Young Adults in Japan.

Age (years)	Most common	Second most common	Third most common	Fourth most common	Fifth most common
15–19	Leukemia (24%)	Germ cell tumor or gonadal tumor (17%)	Lymphoma (13%)	Brain tumor (10%)	Bone tumor (9%)
20–29	Germ cell tumor or gonadal tumor (16%)	Thyroid cancer (12%)	Leukemia (11%)	Lymphoma (10%)	Cervical cancer (9%)
30–39	Female breast cancer (22%)	Cervical cancer (13%)	Germ cell tumor or gonadal tumor (8%)	Thyroid cancer (8%)	Colorectal cancer (8%)

Taken from the National Cancer Research Center Cancer Information Service’s “Cancer Registry and Statistics” https://ganjoho.jp/reg_stat/statistics/stat/child_aya.html

During the period from initial diagnosis to treatment, AYA patients go through various life stages such as school life, higher education, employment, marriage, childbirth, and child-rearing in addition to experiencing significant changes in the personal relationships around them. Psychosocial support for AYA patients is highly individualized and their needs differ depending on the period in their life ^(6), (7), (8)^. In addition, the degree of physical and mental development greatly differs between teenagers and people in their 30s, and there are also large individual differences in development among teenagers. Thus, patients’ decision-making abilities are diverse as well.

The periods when psychosocial support is needed can be roughly divided into three: before and during treatment, after treatment (survivorship), and end-of-life. Before and during treatment period, providing information from a long-term perspective such as how treatment will affect the career plan of that AYA patient, types of long-term side effects, and how long will they last are important. Insufficient prior information about treatment may deteriorate treatment adherence and prognosis ^(9)^. In particular, information regarding fertility should be provided as early as possible before starting treatment ^(10)^. In addition, there are many AYA patients who left their jobs because of their diagnosis and are now in financial distress ^(11)^. Medical staff try not to leave out any needs patients might have related to hospital visits or daily life during recuperation. After treatment, for AYA cancer survivors, long-term side effects may persist and there is inadequate information and support regarding how to provide care and psychosocial support for these patients ^(12), (13), (14), (15), (16), (17), (18), (19)^. Having a survival care plan helps to reduce this shortage of information for post-treatment AYA patients ^(20)^. In addition, since physical and psychological long-term side effects have a great impact on employment ^(21)^, this has to be considered as well. End-of-life stage AYA patients are more likely to receive intensive care and the quality of end-of-life care is low ^(22), (23), (24)^. Although it is not easy to discuss end-of-life topics with young people ^(25), (26)^, patients wish to know their prognosis, use palliative care service, and stay home at end of life ^(27)^. Hence, end-of-life discussion should be started from the time when the patient’s general condition is stable, rather than in life-threatening situation; ideally, this should be made by not just the patient themselves but with the input of their family and doctors as well ^(28)^. Programs such as Five Wishes and Voicing My CHOiCES™ are available tools to support the drafting of advance directives for AYA patients ^(29)^.

To provide psychosocial support for AYA patients, certain skills are required of healthcare professionals (HCPs), and for patients, there has to be an understanding of barriers that might impact their care ^(30)^. While HCPs are highly aware of insurance and financial issues and long-term side effects after treatment, they are not aware of problems related to friendship ^(31)^, employment, education, health behaviors, sexuality ^(17), (32)^, and social and family issues ^(33)^; as a result, they do not adequately provide information about these topics. In addition, access to medical resources, especially the elimination of barriers to participation in clinical trials, is an issue that needs to be addressed ^(34)^. Some tools to support these issues are available ^(35), (36), (37)^. Some have attempted to use digital tools ^(38), (39)^, which are suitable for AYA patients, who use social networking services and the internet to collect information ^(40)^.

Thus, there are many problems in providing psychosocial supports to AYA patients with cancer. This has been recognized by the Japanese policymakers, and to enrich care for AYA patients was incorporated in the Third Cancer Control Program 2017 ^(41)^. In that program, three policies were described: establishing framework of medical service for AYA patients, aggregating institutions that can provide various psychosocial supports for AYA patients, and developing logistics for giving information and providing reproductive medicine.

In this review article, the process for developing the AYA support team (AST) in National Cancer Center Hospital will be illustrated.

## The AYA Support Team

Medical care and support for AYA patients can be psychologically and socially demanding. This is because it involves dealing with a variety of diseases and the diverse needs that significantly change from childhood to adulthood. In our institution, as many as 590 new patients in AYA generation appeared in 2019. This was about 4.5% of overall new patients of that year. An interdepartmental team is required to provide high-quality support. The team can standardize the treatment and quality of care, accumulate experience on highly individualized care, and share know-how and best practices among HCPs.

**Figure 1.**
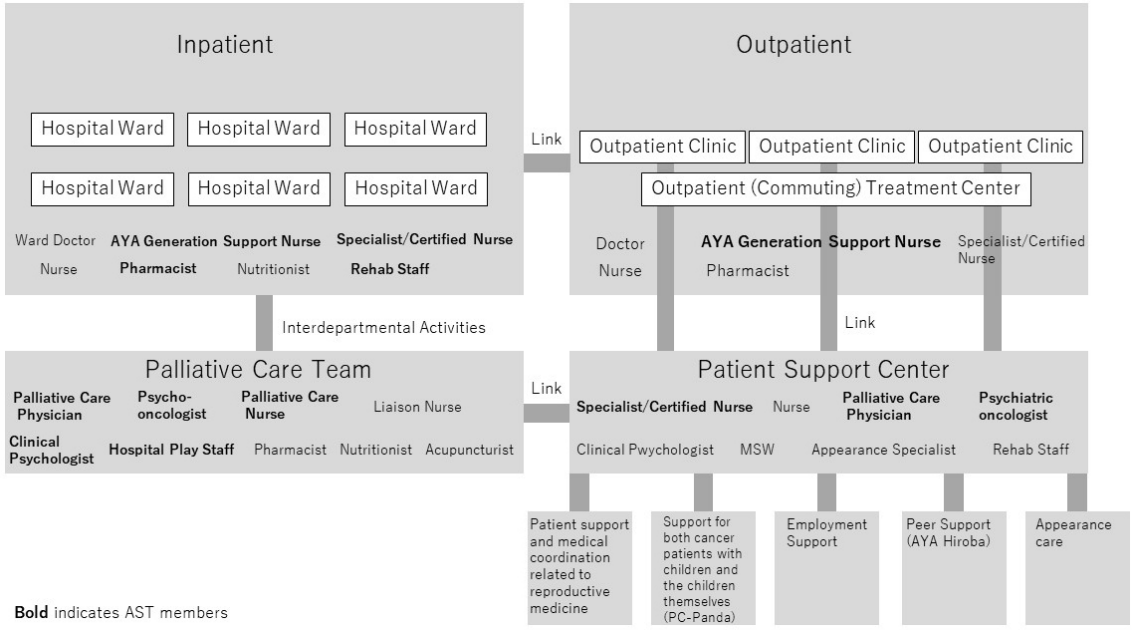
Outline of the adolescents and young adults support team.

HCPs involved in the cancer treatment of AYA patients at our institution make up the AST. The patient support center (PSC), which is an outpatient department where patients can consult about various problems, and the palliative care team (PCT), which is an interdepartmental team involved in cancer treatment of inpatients, consists of the main framework of the AST (Figure 1). The AST systematically finds patients in need of assistance and offers them support as a multidisciplinary team. In addition, the AST also provides specialized services such as patient support and medical coordination related to reproductive medicine (reproductive health support team), supporting cancer patients with children and the children themselves (PC-Panda) and offering employment support and peer support (AYA Hiroba).

## 1. AST Launch Process (Figure 2)

**Figure 2.**
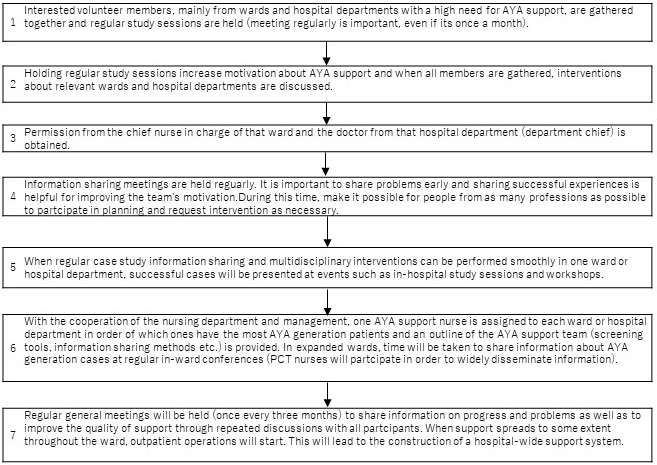
Adolescents and young adults support team launch process.

Each AYA patient has different, diverse needs. Therefore, AST needs to use an efficient, comprehensive team approach. In the past, there have been reports from several institutions and communities regarding attempts to build a framework to support AYA patients ^(42), (43), (44), (45), (46)^. According to these reports, psychosocial support of AYA patients can be divided into three categories: (1) providing integrated programs, (2) strengthening and providing individual support systems, and (3) introducing tools for decision-making support and screening. Supporting AYA patients requires a variety of approaches from different perspectives and so requires variety of HCPs.

### Deciding team membership

#### ①From the oncology department

In addition to the core members, who are doctors, nurses, and pharmacists from hospital departments where there are many opportunities to treat AYA patients (Department of Oncology, Bone and Soft Tissue and Pediatric Oncology).

#### ②From an interdepartmental healthcare team

An interdepartmental healthcare team (PCT, nutrition support team) picks up AYA patients and then starts support activities mainly aimed toward inpatients.

The AST began with a multidisciplinary hospital ward conference in the Department of Breast Oncology (①). PCT and PSC then joined the team, which strengthened the in-hospital interdepartmental activity system (②). Next, an in-charge AYA nurse was nominated in each hospital ward that had many AYA patients. Every 3 months, an AST general conference was held to share the support status of inpatients. After AST activities spread through all wards, support was then expanded to the outpatient department.

### Deciding activities

Medical resources that are available in different facilities and areas vary ^(47)^ and activities are determined according to what resources are accessible. AST discovers and appropriately refers AYA patients in need of assistance to professionals through activities such as the following: presenting case studies at conferences, holding study sessions for staff, disseminating information about the AST’s workflow throughout the hospital, managing the screening implementation system, and operating patient salons. Occasionally, young healthcare professionals caring for patients of similar age to themselves are at a risk of burnout ^(48)^. AST also takes note of the presence of such medical personnel within the healthcare team.

### Requesting approval from hospital executives as an in-hospital organization

Approval from hospital management for AST activities to be recognized as official needs to be obtained. Currently, in terms of medical fee reimbursements, there are no financial incentives for using AYA support in Japan. However, policymakers are working to develop a medical care system that can respond to the needs of AYA patients according to their individual situations as part of domestic cancer policy (Cancer Control Act and Cancer Control Programs) ^(41)^.

### Appealing to the hospital

In the early stages of launching an AST, it is often an initiative that is spearheaded by a limited number of hospital wards and departments. Knowledge of these activities is then spread throughout the hospital, and more colleagues who will help to jointly support AYA patients are recruited.

### Improve activities using the PDCA (Plan-Do-Check-Act) cycle

Activities are reviewed regularly from the patients’ point of view in order to make improvements. In addition, by reviewing team management regularly, how we look at patients is adjusted and the medical care system is modified accordingly. During the weekly AYA conference, information about AYA patients is shared between members with different occupations. At the AST general conference that is held once every 3 months, the current activities of the AST are shared and members of each occupation discuss what is going well and what needs to be improved about the activities they are taking part it.

## 2. The Role of Each Occupation of the AST

Treatment of AYA patients is often long-term and highly invasive. Successful treatment requires not only the management of adverse events but also understanding changes in appearance, changes in relationships with family and friends, daily life guidance (diet, exercise), financial toxicity, interruption of schooling and career, and psychosocial factors such as love, marriage, and pregnancy ^(8), (25), (33), (49), (50), (51), (52)^. Dealing with these requires information sharing and collaboration among members of the healthcare team.

### Nurse in charge of the patient

Being in a position that is closest to the patient, they establish a relationship with, talk to, become emotionally close with, accept, and elicit the needs of the patient. The AYA nurse in each department keeps track of the AYA patient’s visitation schedule and manages the screening process.

### Certified nurse/certified nurse specialist

They provide more specialized, comprehensive support for AYA patients and their families and supporters ^(53)^. In addition, they also provide logistic support, reduce psychological burden, and offer support methods to nurses as a consultant and an educator.

### Attending physician (Oncologist)

They determine treatment policy and provide treatment to AYA patients. Social background information shared within the AST is important for determining treatment policies. Some patients can make decisions by themselves, but many want to take part in shared decision-making with their families and healthcare professionals ^(54)^. Parents play an important role in the decision-making of AYA patients aged 15 to 20 years ^(55)^. When important information is being communicated to the AYA patients, such as curability, the effect on fertility and appearance, family members, and other people close to them should attend these discussions. Therefore, they coordinate shared decision-making process with not only the patients but also parents, family members, other people close to the patients, and healthcare professionals.

### Palliative care physician

Palliative care physicians mainly share the course of treatment with patients and their families, which includes the prevention, prediction, and alleviation of physical distress; have a comprehensive understanding of the physical, mental, social, and spiritual aspects of the patient; and support advance care planning. Discussions about death are painful for both the medical staff and the patient’s family ^(25)^. They talk to the patient and their families about the terminal stages of the disease and support the decision-making process, with emphasis on what is important to the AYA patient and in the event of an emergency, what are the patient’s wishes.

### Psycho-oncologist

Psycho-oncologists mainly have the following five roles: ①supporting the transition period from childhood to adulthood, ②dealing with issues unique to the AYA generation, ③understanding and solving problems by having a bird’s-eye view of the functioning and difficulties of the entire healthcare team, ④coordinating communication between medical professionals, and ⑤preventing staff burnout and investigating the reason behind the maladaptive behaviors of patients (being the fault of the family or medical staff, refusal of care, low motivation for treatment).

### Pharmacist

Pharmacists explain the patient’s treatment schedule, the side effects of anticancer drugs, and how to counteract those side effects. Pharmacists are often consulted about the effects of treatment on daily life and fertility, and by providing guidance about medications, they gather information from the patient about problems related to topics such as taking exams, job hunting, friendships, marriage, children, partners, parents and siblings, work, and medical expenses.

### Registered dietitian

Registered dieticians manage nutrition and diet as a part of treatment and care.

Patient need for nutrition is high ^(8)^. Many patients receive complementary and alternative therapies during treatment ^(56)^, some of which are expensive and adversely affect treatment. So they also instruct patients not to be confused by incorrect information.

### Rehabilitation staff (physical therapist, occupational therapist, speech therapist)

Rehab staff members help patients maintain and restore physical function during treatment and prevent cancer-induced disuse syndrome. For AYA patients, physical activity is important in many ways, including maintaining physical function, leisure and hobbies, and competition ^(53)^; patient need for physical activity is also high. For AYA patients, returning to their pretreatment environment after treatment is often challenging. Rehabilitation staff evaluate physical function and create and implement rehabilitation plans tailored to each patient.

### Medical social worker (MSW)

MSWs support AYA patients by aiding with financial problems, utilizing social resources and systems, adjusting the patient’s recovery environment, and helping with finding employment and enrolling in school. Many of the difficulties patients experience during treatment are due to social systems. The AYA generation may have little knowledge of social systems, and MSWs provide information and support not only to the patients but also to their families.

### Clinical psychologist

The AYA generation is in the developmental stage from adolescence to young adulthood, so there are large individual differences in mental development. Therefore, both the chronological and mental ages are evaluated and patient care is individualized. In addition, the degree of social and mental independence greatly varies between individuals, and the relationships between patients and their families are also diverse. Clinical psychologist evaluates both these issues and family dynamics and notes who the decision-maker is and how patient-family relationships affect treatment and recovery.

### Appearance care specialist

The appearance of the patient changes in various ways due to the cancer or treatment, such as hair loss, changes in pigment, and loss or deformation of body parts due to surgery. Even if these changes do not affect the patient’s health prognosis, they have a significant effect on their quality of life. Appearance care is a method for improving QOL by alleviating the pain caused by changes in appearance using medical, cosmetic, and psychosocial means. A clinical psychologist with expertise and skills in cosmetics and beauty aims to comprehensively reduce pain by using an approach that also incorporates aspects such as patient cognition and communication rather than simply improving changes in appearance cosmetically.

### Hospital play staff (HPS/child life specialist/child care supporter)

HPS mainly have the following four roles: ①understanding changes in the patient’s medical condition and changes in their living situation that affects childcare and evaluating the reason behind the patient’s concerns, ②evaluating child development and giving advice to parents, ③providing information related to patient group networks and social support services for childcare, and ④encouraging strong relationships so that communication between the visiting children and the parents can deepen and a feeling of mutual love can be felt.

Through the above activities, psychosocial supports are provided for AYA patients and their spouses and children. They also advise the healthcare team on how to relate to patients and their children.

## 3. The Unmet Needs of the AYA Generation and Screening for Those Needs

### Screening methods and tools

**Figure 3.**
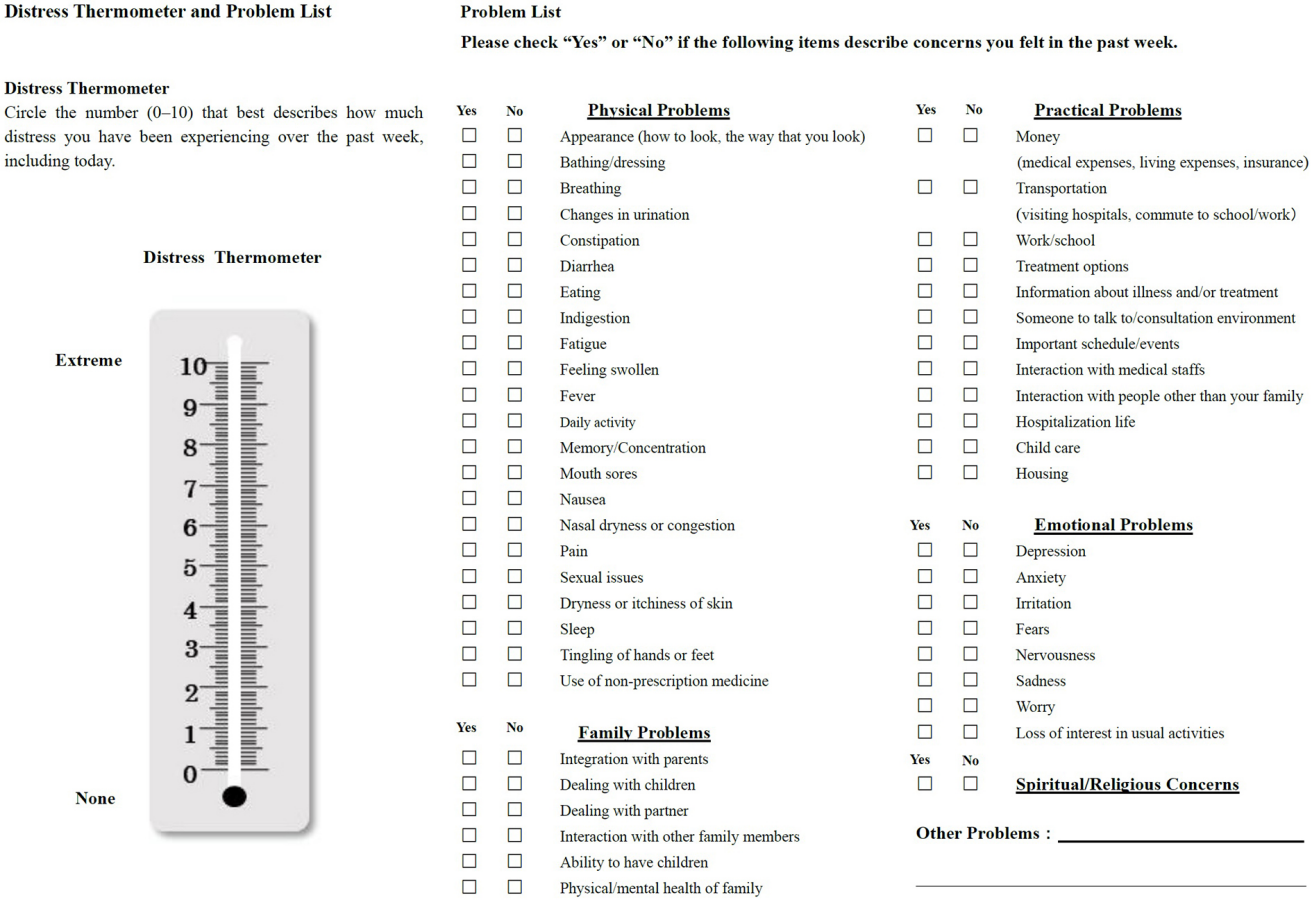
Established screening tool.

It has been reported that screening is useful for identifying various unmet needs at an early stage among AYA generation patients and implementing a multidisciplinary approach ^(57)^. By using a screening tool (ST), it is possible to systematically gather information about the needs of patients. Since patient needs include financial and sexual issues that are difficult to talk about directly, STs can help medical staff collect required information easier in a standardized way. The AST created its own ST based on the ST introduced in the NCCN guidelines (Figure 3) ^(58)^. This ST is comprised of a checklist of 49 items: a pain thermometer, which expresses the degree of pain, and physical problems (22 items), family problems (6 items), daily life problems (12 items), emotional problems (8 items), and spiritual or religious problems (1 item). Support is then provided according to the flowchart, which is based on the checked items.

### Screening in clinical settings

#### Inpatient

The nurse in charge of the patient performs a screening using the ST on the day the patient is admitted. Information for the ST can be written by hand or using a tablet and then that information is recorded in the electronic medical record. The nurse then goes through the checklist and checks applicable items while listening to the patient. If there is a previous ST, the current one is compared to it, and if there are any discrepancies in the checked items, they listen carefully to the information being provided by the patient. Patient information is then shared at a weekly, multidisciplinary healthcare team conference in the ward and the patient’s need for support is evaluated.

#### Outpatient

The nurse in charge of AYA support first gathers information about the AYA patient’s outpatient visit schedule. The patient is asked to fill out the ST while the patient is waiting for their outpatient medical examination by the nurse in charge of that patient. After the screening, they inform the nurse in charge on the subsequent outpatient visit days of the details of that day’s screening and the next screening time.

Before a definitive cancer diagnosis, the treatment of some patients may be unclear. For these patients, even after screening, pinpointing specific issues may be difficult. It is easier to understand patient needs if screening is performed after a confirmed diagnosis and the treatment has already been determined. Therefore, it is important to understand the examination and treatment schedule from the first visit and perform a screening at an appropriate time. After confirming whether there are any problems such as those concerning fertility, worries about children, or need for employment support, patients are promptly introduced to a specialist.

Regardless of whether it is during inpatient or outpatient care, when screening is conducted anywhere in the hospital, patient information is shared between inpatient and outpatient care facilities using electronic medical records so that they can be linked seamlessly.

#### Outpatient chemotherapy room

In addition to at the start of treatment and immediately before the end of treatment, screening is performed about every 3 months according to the treatment evaluation.

### Screening for the support needs of specific patients

#### Pediatric tumors (15-18 years old)

At the time of initial hospitalization, clinical psychologists get information from patients and their families. Nurses also initiate screening procedures using the ST. Thereafter, screening will be conducted at every admission and patients and their families will be referred to medical specialists according to their needs. It is often difficult to adequately illicit information from adolescent patients because of their unique developmental characteristics, which may not allow them to express their feelings and concerns to the adults who just met for the first time. Therefore, it is crucial to create a relationship dynamic that makes it easier for patients to express their thoughts in addition to utilizing the ST.

#### Hematological malignancies

As hematological malignancies often progress rapidly, patients’ needs, especially that of reproductive function, should be caught by HCPs as soon as possible. Screening is performed in an outpatient facility between the first visit and the start of treatment. At the time of the first visit, there is little information about the patient and treatment. Therefore, first, a HCP listens to the thoughts of the patient and their families and related information may also be provided as needed. After that, when diagnosis and treatment details are decided, a formal screening is performed pretreatment. Before treatment starts and at the start of the first treatment, patients and their families are dealing with many problems in the chaos of the recent cancer diagnosis, so the screening is conducted within a short interval of time. Immediately before the end of treatment, HCPs explore the new, post-treatment rehabilitation needs of the patient using screening. After treatment is completed, screening is continuously conducted during the period when the therapeutic effect of treatment is being determined.

## 4. AST Provided Specialist Support Services

### Fertility consultation and support (reproductive health support team)

**Figure 4.**
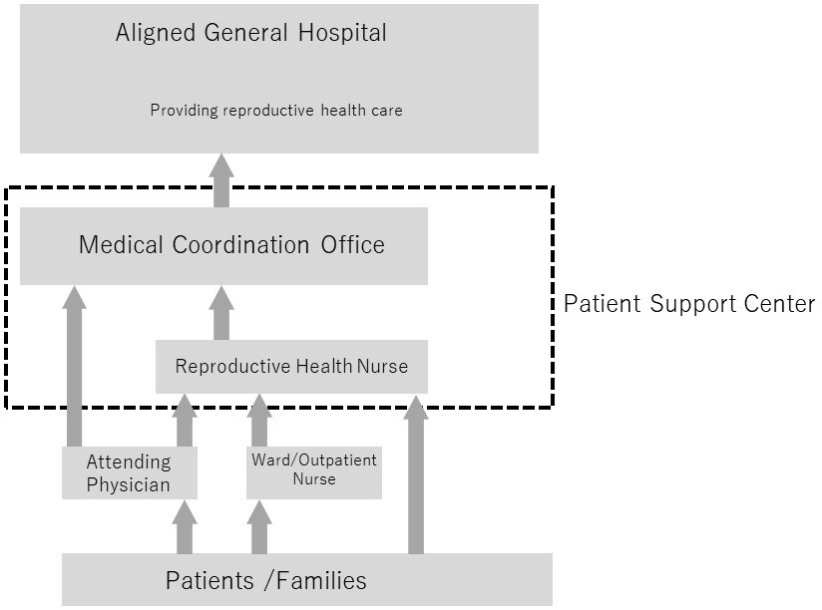
Outline of the fertility consultation and support.

Since there is not a reproductive medicine department at the cancer center, support such as consultations and medical care are provided in cooperation with nearby medical institutions that can offer these services (St. Luke’s International Hospital’s Reproductive Medical Center, etc.) (Figure 4). To smoothly carry out the decision-making process regarding fertility preservation intervention in the short pre-cancer treatment time period, a consultation desk was set up at the PSC and an inter-facility information sharing system was built.

Patients and their families as well as from medical staff can consult about fertility issues including fertility preservation, the timing of pregnancy and childbirth during and after treatment, fertility evaluation, communication with partners, and the foster parent system. For teenage patients, information is provided to the parents or guardians as well as to the patient.

### Child support (multidisciplinary support team for cancer patients and families with minors: PC-Panda)

One in four inpatients at our hospital has a child ^(59)^ and is being treated while raising children. AYA patients often worry about separation from their children in decision-making on their treatment ^(60)^. The multidisciplinary support team “PC-Panda” (Parents with cancer and Children support --Professionals and associates) consists of HPS, CLS, and PCT physicians, clinical psychologists, and nurses. This team provides care to patients and their families with the following aspects: “coping with the anxiety of cancer patients and families with minor children,” “promoting better communication within these families,” and “thinking of ways to create openness in families” with five stages: information, screening, indirect support, direct support for adults, and direct support for children (Table 2).

**Table 2.** Steps of PC-Panda Support.

Step 1	Information	Distribute information about PC-Panda through the in-hospital bulletin board and patient education classes
Step 2	Screening	Check the medical records of inpatients aged 20 to 50 years and AYA patient screening results
Step 3	Indirect support	Check for concerns in children and provide information using leaflets and booklets
Step 4	Direct support for adults	Conduct individual interviews with patients and their families
Step 5	Direct support for children	Help children increase their ability to cope with their situation and vocalizing their feelings

Abbreviations: AYA: Adolescents AND Young Adults, PC-Panda: Parents with Cancer and Children Support Professionals and Associates

### Employment support

In this hospital, AYA patients account for 22% of employment counseling. Employment support for AYA patients has three points: (1) first-time job hunting, (2) returning to work and reemployment, and (3) continuation of work after returning to work. At the PSC, in addition to the employment support offered by MSWs, social insurance labor consultants and Hello Work (public employment security office) staff hold individual consultations once a week to provide employment and work-life balance support for patients.

### Peer support (AYA Hiroba)

AYA patients tended to be isolated because there was no opportunity for patients to meet other patients of the same age and discuss their concerns or exchange information in the hospital. The “AYA Hiroba (a get-together for AYA patients)” was opened in 2016 and is held once a month in the hospital for AYA patients. Two HCPs from the AST facilitate it and participants freely choose topics they want to discuss and exchange information. In situations where face-to-face meetings are difficult due to the spread of the novel coronavirus infection, an online conference system is used to hold web meetings. This makes it possible for AYA patients to participate in the event without actually visiting the hospital.

## Conclusions

The AST has built a screening system and through the AYA conference held once a week and the AST general conference held once every 3 months, patient information and needs as well as information on AST operations are shared among the multidisciplinary healthcare team; the most notable feature is that this system can flexibly provide various psychosocial support services. Future tasks include also addressing the needs of survivors during follow-up and providing them with psychosocial support ^(19), (61)^, supporting those left behind, including parents and siblings, introducing more potential participants to ongoing clinical trials and promoting research related to AYA support.

## Article Information

### Conflicts of Interest

None

### Acknowledgement

This manuscript was supported by the National Cancer Center Research and Development Fund (grant number 30‐A‐13).

### Author Contributions

All authors conceptualized the study. HI took the lead in drafting the manuscript. HI and TH drafted the initial summary of the findings. ES supervised the findings and writing of this work. All authors discussed the results, provided critical feedback, and reviewed the manuscript. All authors approved the final version of the manuscript.
